# Bone Marrow Metabolism Is Impaired in Insulin Resistance and Improves After Exercise Training

**DOI:** 10.1210/clinem/dgaa516

**Published:** 2020-08-12

**Authors:** Ronja Ojala, Kumail K Motiani, Kaisa K Ivaska, Milja Arponen, Jari-Joonas Eskelinen, Kirsi A Virtanen, Eliisa Löyttyniemi, Marja A Heiskanen, Mueez U-Din, Pirjo Nuutila, Kari K Kalliokoski, Jarna C Hannukainen

**Affiliations:** 1 Turku PET Centre, University of Turku, Turku, Finland; 2 Institute of Biomedicine, University of Turku, Turku, Finland; 3 Turku PET Centre, Turku University Hospital, Turku, Finland; 4 Department of Biostatistics, University of Turku, Turku, Finland; 5 Department of Endocrinology, Turku University Hospital, Turku, Finland

**Keywords:** Bone marrow, positron emission tomography, exercise training, metabolism, osteocalcin, PINP

## Abstract

**Context:**

Exercise training improves bone mineral density, but little is known about the effects of training on bone marrow (BM) metabolism. BM insulin sensitivity has been suggested to play an important role in bone health and whole-body insulin sensitivity.

**Objective:**

To study the effects of exercise training on BM metabolism.

**Design:**

Randomized controlled trial.

**Setting:**

Clinical research center.

**Participants:**

Sedentary healthy (n = 28, 40–55 years, all males) and insulin resistant (IR) subjects (n = 26, 43–55 years, males/females 16/10)

**Intervention:**

Two weeks of sprint interval training or moderate-intensity continuous training

**Main outcome measures:**

We measured femoral, lumbar, and thoracic BM insulin-stimulated glucose uptake (GU) and fasting free fatty acid uptake (FFAU) using positron-emission tomography and bone turnover markers from plasma.

**Results:**

At baseline, GU was highest in lumbar, followed by thoracic, and lowest in femoral BM (all *P*s < 0.0001). FFAU was higher in lumbar and thoracic than femoral BM (both *P*s < 0.0001). BM FFAU and femoral BM GU were higher in healthy compared to IR men and in females compared to males (all *P*s < 0.05). Training increased femoral BM GU similarly in all groups and decreased lumbar BM FFAU in males (all *P*s < 0.05). Osteocalcin and PINP were lower in IR than healthy men and correlated positively with femoral BM GU and glycemic status (all *P*s < 0.05).

**Conclusions:**

BM metabolism differs regarding anatomical location. Short-term training improves BM GU and FFAU in healthy and IR subjects. Bone turnover rate is decreased in insulin resistance and associates positively with BM metabolism and glycemic control.

**Clinical Trial Registration Number:**

NCT01344928.

Bone marrow is the porous tissue found within all bones of the body. Bone marrow consists of hematopoietic cells (blood cells and their precursors), other stem cells, adipose tissue, and trabecular bone. The ratio of these components differs according to anatomical location. When a child is born, almost all their bone cavities are filled with hematopoietic bone marrow tissue. During aging, hematopoietic bone marrow is slowly replaced by adipose tissue from the periphery toward the axial skeleton (1). In adults, hematopoietic bone marrow can still be found in the axial skeleton and in the proximal ends of long bones, such as femur and humerus (2). Thus, one important function of bone marrow in the axial skeleton is the production of blood cells while the bone cavity of long bones serves as a specialized fat depot. However, despite the known differences in bone marrow, it is unclear whether the metabolism differs between anatomical locations.

Bone marrow cavity is the only place in the human body where bone and fat tissue are directly connected without any membrane between the 2 tissues (3). It is not known whether bone marrow metabolism affects bone turnover and homeostasis. For example, bone marrow insulin resistance (IR) may be a potential factor for impaired bone health. Most studies have found increased risk of bone fractures in type 2 diabetic patients, despite normal or increased bone mineral density (4-6). It has been suggested that increased bone marrow adipose tissue volume is associated with increased fracture risk (7,8). Using magnetic resonance spectroscopy, Schellinger et al found an inverse relationship between bone marrow adipose tissue volume and bone integrity (9). Furthermore, the volume of bone marrow adipose tissue correlates negatively with hematopoietic activity of bone marrow (10).

It is known that exercise training improves whole-body insulin sensitivity. To our knowledge, only Huovinen et al have studied the effects of exercise training on bone marrow insulin sensitivity. It was shown that 4 months of resistance training increased femoral bone marrow insulin sensitivity in elderly female subjects (11). However, it is not known whether bone marrow metabolism is impaired in IR and whether exercise training can improve it.

We set out to investigate the short-term effects of two training methods, sprint interval training (SIT) and moderate-intensity continuous training (MICT), on glucose and free fatty acid metabolism of bone marrow in healthy and insulin resistant subjects using 2-[^18^F]fluoro-2-deoxy-D-glucose (^18^F-FDG) positron emission tomography (PET) and 14(R,S)-[^18^F]fluoro-6-thia-heptadecanoic acid (^18^F-FTHA) PET imaging. The aims of our study were to investigate bone marrow insulin-stimulated glucose uptake (GU) and fasting free fatty acid uptake (FFAU)

- at baseline between
  - healthy and insulin-resistant men and
  - insulin-resistant men and women.
- after a two-week training intervention between
  - healthy and insulin-resistant men,
  - insulin-resistant men and women, and
  - between SIT and MICT training modes.

In addition to PET imaging, bone turnover markers were measured from plasma. We hypothesized that glucose and fatty acid uptake would be highest in the lumbar vertebral region due to hematopoiesis, impaired in IR, and lower in males compared to females. We further hypothesized that exercise training would effectively enhance bone marrow metabolism, with SIT being superior to MICT.

## Methods

### Ethics

This study was part of a larger study entitled “The Effects of Short-Time High-Intensity Interval Training on Tissue Glucose and Fat Metabolism in Healthy Subjects and in Patients with Type 2 Diabetes” (NCT01344928). Basic outcomes included in this article have already been published for some of the study population (whole-body insulin sensitivity, aerobic fitness, and basic characteristics) (12-15). No data considering metabolism of bone marrow have previously been published from this study. The study was conducted at Turku PET Centre (University of Turku, Turku, Finland), Turku University Hospital (Turku, Finland), and the Paavo Nurmi Centre (Turku, Finland) between March 2011 and September 2015 in compliance with the Declaration of Helsinki. The study protocol was approved by the ethical committee of the Hospital District of Southwest Finland (decision 95/180/2010 §228). Before any measurements were performed the purpose and potential risks of the study were explained and written consent was obtained.

### Subjects

Middle-aged, sedentary, healthy subjects and subjects with IR were recruited for the study via newspaper advertisements, personal contacts, and traditional and electronic bulletin boards. The inclusion criteria for healthy subjects (n = 28, aged 40-55 years, all male) were as previously published by Honkala et al (12).

**Figure 1.**
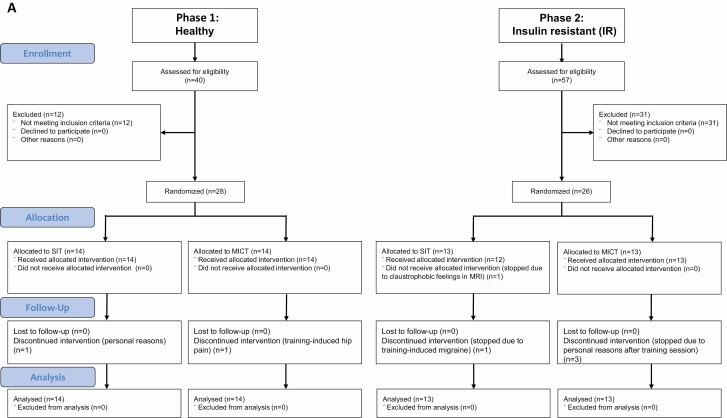

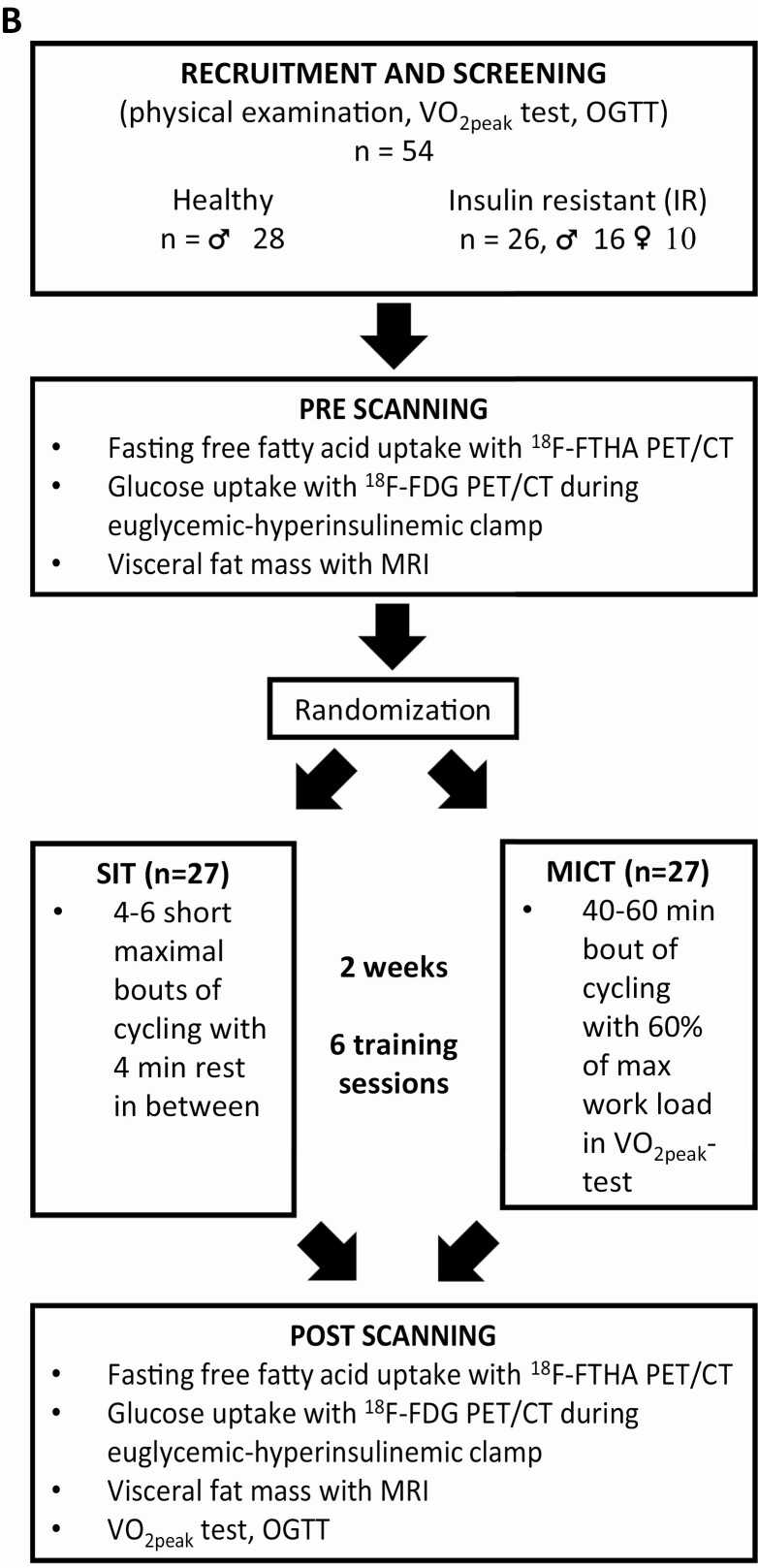
(A) CONSORT flow diagram. The analyses were carried out using the intention-to-treat principle and included all the randomized participants. (B) Study design. Abbreviations: ^18^F-FDG, 2-[^18^F]fluoro-2-deoxy-D-glucose; ^18^F-FTHA, 14(R,S)-[^18^F]fluoro-6-thia-heptadecanoic acid; MICT, moderate-intensity continuous training; MRI, magnetic resonance imaging; OGTT, oral glucose tolerance test; SIT, sprint-interval training; VO_2peak_ test, aerobic capacity;

Inclusion criteria for IR subjects (n = 26, aged 43-55 years, male/female = 16/10) were as previously published (14). Of the 26 IR subjects 17 (11 men) met the criteria for type 2 diabetes mellitus (T2DM) and 9 (5 men) met the criteria for prediabetes having impaired fasting glucose concentrations and/or impaired glucose tolerance as defined by American Diabetes Association guidelines (16). Of the 17 subjects with T2DM, 13 were treated with at least 1 type of oral hypoglycemic medication. The median diabetes duration was 4.2 years. Four subjects (1 man) met the criteria for T2DM at screening and had no previous medication. In addition, 7 IR subjects were taking statins. In total, 7 subjects dropped out during the intervention, 1 due to exercise-induced hip pain, 1 due to training induced migraine, 1 due to claustrophobic feeling within the magnetic resonance imaging (MRI) scanner, and 4 due to personal reasons (Fig. 1A). All participants were asked not to change their habitual dietary intake during the study period.

### Study design

Study design is shown in Fig. 1B. Initial screening consisted of a physical examination, an oral glucose tolerance test (OGTT), and a VO_2peak_ test to assess the participant’s glycemic status and aerobic capacity. At least 1 week after the screening day ^18^F-FTHA-PET study was performed to measure FFAU in thoracic vertebral, lumbar vertebral, and femoral bone marrow. The following day, ^18^F-FDG-PET study was performed during euglycemic-hyperinsulinemic clamp to measure whole body insulin sensitivity (M-value) and GU in bone marrow in the same anatomical regions. Visceral fat was measured with MRI as previously described by Motiani et al (17). Subjects were asked to avoid exhausting exercise and caffeinated and alcoholic beverages and to stop all antidiabetic medications 48 h prior to any measurements.

After the pretraining measurements the subjects were randomized into 2 training groups for the 2-week exercise intervention, SIT and MICT, as previously described (12). The final group sizes for healthy subjects were n = 14 for SIT and n = 14 for MICT, and for IR subjects, n = 13 for SIT and n = 13 for MICT.

After the training intervention, all measurements were repeated starting ~48 h after the last training session. ^18^F-FTHA-PET study was performed first. The following day, ~72 h after the last training session, ^18^F-FDG-PET study was performed. Finally, OGTT and VO_2peak_ test were repeated after ~96 h after the last training session.

### Exercise intervention

The intervention was carried out as previously described by Honkala et al (12). Both training groups had six supervised training sessions within two weeks in controlled laboratory conditions. Given the nature of the intervention, no blinding was used. The SIT sessions consisted of 4–6 maximal all-out cycling bouts (Monark Ergomedic 894E; MONARK, Vnasbro, Sweden) of 30 s with a 4-min recovery period in between (Wingate protocol). During the recovery period, the subjects could either remain still or do unloaded cycling. The amount of cycling bouts started at 4 and was increased by 1 bout after every other training session. The study subjects were familiarized with the SIT protocol ~1 week before the intervention by doing two 30-second bouts. The MICT sessions consisted of 40 to 60 min of cycling at a moderate intensity with a load of 60% of their individual VO_2peak_ intensity (Tunturi E85; Tunturi Fitness, Almere, Netherlands). The cycling time started at 40 min and was increased by 10 min every other training session until 60 min was reached.

### PET measurements and euglycemic-hyperinsulinemic clamp

The PET/computed tomography (CT) images were acquired using GE Discovery TM ST System (General Electric Medical Systems, Milwaukee, WI, US). CT images were acquired for anatomical reference and radiodensity extraction. The participants fasted for ≥10 h before the PET studies. To arterialize venous blood for the length of the study, an electrically powered heating cushion was placed under the arm where the blood samples were taken from.

Bone marrow FFAU was measured using ^18^F-FTHA in a fasting state. Lumbar (vertebrae Th12-L3), femoral (middle of the thigh), and thoracic regions (vertebrae Th1-Th4) were then scanned starting at ~46, ~65 and ~86 min after tracer injection (156 [SEM 1.1] MBq), respectively.

Glucose uptake was measured using ^18^F-FDG during euglycemic-hyperinsulinemic clamp. The clamp was performed as previously published by Defronzo et al (18). Whole-body insulin-stimulated GU (M-value) was calculated from the glucose infusion rate as described earlier (17). The ^18^F-FDG-PET study (157 [SEM 0.9] MBq) started 91 min (SE 2) after the start of the clamp and lumbar vertebral, femoral, and thoracic vertebral regions were scanned starting ~47, ~67, and ~93 min after tracer injection, respectively. In the IR group, thoracic region was not scanned.

### Image analysis

**Figure 2.**
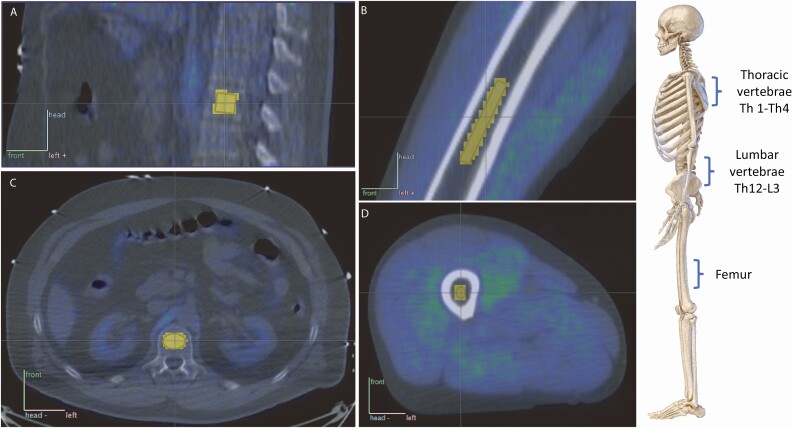
An example of the shape and positioning of the region of interest (ROI) from which time activity curves were extracted. Sagittal PET/CT image of lumbar vertebral (A) and femoral (B) regions. Transaxial PET/CT image of lumbar vertebral (C) and femoral (D) regions. CT scans were used as anatomical reference.

The imaging data obtained from the PET scanner were corrected for dead-time, decay, and photon attenuation. The 3D ordered subsets expectation-maximization method was used to reconstruct the images. Carimas 2.9 software (http://turkupetcentre.fi) was used to manually draw 3-dimensional regions of interest (ROIs) in the marrow cavities of femurs and thoracic and lumbar vertebrae. CT images were used as anatomical reference. The ROIs were carefully drawn to only include the marrow cavity and to leave out cortical bone and surrounding tissue. An example of the shape and positioning of the ROIs can be seen in Fig. 2. From these ROIs, time activity curves were extracted.

The radiodensity of tissue is expressed in Hounsfield units (HU), obtained from a linear transformation of attenuation coefficients based on the arbitrary definitions of air (−1000 HU) and water (0 HU). On this scale, fat has a density of −60 to −120 HU (19). In bone marrow, the amount of fat cannot be quantified, but the lower the HU, the higher the fat content. For radiodensity analysis, ROIs were drawn onto the CT images. In the thigh area, a ROI covering the entire mid-shaft of the femur was drawn. The CT voxels within this ROI were then thresholded to separate cortical bone from the bone marrow tissue. The HU threshold level for differentiating cortical bone from bone marrow tissue was considered to be 400 HU based on visual evaluation as well as previously documented HU range of cortical bone and bone marrow (20). In lumbar area, ROIs were drawn onto the CT images carefully avoiding cortical bone as in PET ROIs. No thresholding was necessary, as only trabecular bone area was included.

### Other measurements: VO_**2peak**_test, OGTT, and bioimpedance analysis

Aerobic capacity was determined by performing an incremental VO_2peak_ bicycle ergometer test as previously described by Kiviniemi et al (21). A 2-h, 75-g OGTT was done after the subjects had fasted for at least 12 h. After ingestion of glucose, blood samples were collected at 0, 15, 30, 60, 90, and 120 min to determine glucose and insulin concentrations in the blood. Body composition was measured with a bioimpedance monitor (InBody 720, Mega Electronics, Kuopio, Finland).

### Bone turnover markers

Blood samples were collected in the mornings of the ^18^F-FTHA-PET studies after an overnight fast and ethylenediamine tetra-acetate plasma samples were stored as aliquots at −80°C. Bone formation was assessed by measuring intact N-terminal propeptides of type I collagen (PINP) (22) by using IDS-iSYS Intact PINP assay (IDS Ltd, UK). Bone-specific osteocalcin, a marker of bone remodeling, was measured with 2-site immunoassay using a previously described protocol (23). Assay detects total osteocalcin and is based on monoclonal antibodies 2H9 and 6F9.

### Statistics

Sample size was calculated for the whole study based on its primary outcome (skeletal muscle GU) (13). No sample size calculation was performed on the outcome measures of this study.

The normal distribution of the variables was tested using Shapiro-Wilk test and evaluated visually. Logarithmic (log_10_) transformations were performed to fulfill the normal distribution assumption (whole-body insulin sensitivity [M-value], lumbar bone marrow FFAU, osteocalcin [for comparisons in insulin resistant group], PINP). Statistical analyses were performed using hierarchical mixed linear models with compound symmetry covariance structure. First, the differences between healthy and IR men were studied with the model, which included 1 within-factor term (time; indicating the overall mean change between baseline and measurement after the intervention), 2 between-factor terms (glycemic status: healthy and IR men; training: SIT and MICT), and 2 interaction terms (time × glycemic status: indicating whether mean change during the study was different between healthy and IR men; time × training: indicating whether mean change during the study was different between SIT and MICT). IR women were only included in comparisons within the IR group to avoid mixing the effects of sex and glucose intolerance. Second, differences between SIT and MICT in IR participants, including both men and women, were studied using a model that included within-factor time (time; indicating the overall mean change between baseline and measurement after the intervention), 2 between-factor terms (training: SIT and MICT; sex: male and female), and 2 interaction term (time × sex: indicating whether mean change during the study was different between IR men and IR women; time × training: indicating whether mean change during the study was different between SIT and MICT). The analyses were carried out using the intention-to-treat principle and included all the randomized participants. Due to the chosen analysis method, also participants with missing data could be included into statistical modelling. Furthermore, model-based means (SAS least square means) and 95% confidence intervals are reported for all the parameters. Correlations were calculated using Pearson’s correlation (Spearman’s rank correlation for nonnormally distributed data).

To study which variables affect the GU and FFAU of bone marrow, we used the multivariate regression analysis, which is a technique that estimates a single regression model with multiple outcome variables and 1 or more predictor variables.

The statistical tests were performed as 2-sided and the level of statistical significance was set at 0.05. The analyses were performed using SAS System, version 9.4 for Windows (SAS Institute, Cary, NC, US).

## Results

Before intervention, IR men had impaired aerobic capacity (*P* < 0.001) compared to healthy men, but training improved aerobic capacity similarly in both groups (time *P* = 0.003, time*IR *P* = 0.23) (Table 1). When divided by training mode, only SIT improved aerobic capacity in IR subjects with no differences between men and women (data not shown). IR men had significantly lower whole-body insulin sensitivity (M-value) at baseline (*P* < 0.001), but it improved after training with no differences between the groups or training modes (time *P* < 0.001) (Table 1). Except for the higher increase in aerobic capacity after SIT, we did not observe any other differences between the training modes in the measured parameters.

**Table 1.** Subject characteristics between healthy and IR men before and after exercise intervention

	Healthy Men		IR Men				
Parameter	Pre	Post	Pre	Post	Baseline	Time	Time*IR
***Anthropometrics***							
Weight (kg)	83.6 [79.7;87.5]	83.3 [79.4;87.2]	96.3 [91.2;101.3]	96.2 [91.0;101.3]	**<0.001**	0.22	0.80
BMI (kg/m^2^)	26.1 [25.1;27.1]	26.0 [25.0;27.0]	30.4 [29.1;31.8]	30.4 [29.0;31.7]	**<0.001**	0.17	0.70
Whole body fat^&^ (%)	22.6 [20.9;24.3]	21.7 [20.0;23.3]	28.8 [26.5;31.2]	28.1 [25.7;30.4]	**<0.001**	**<0.001**	0.78
Visceral fat† (kg)	2.5 [2.0;3.2]	2.4 [1.9;3.08]	4.3 [5.4;3.4]	4.1 [3.1;5.1]	**0.002**	**0.002**	0.48
VO_2peak_ (mL/kg/min)	34.2 [32.7;35.7]	35.7 [34.2;37.2]	29.3 [27.2;31.4]	30.0 [27.9;32.1]	**<0.001**	**0.003**	0.23
***Glucose profile***							
Glucose_fasting_^&^ (mmol/L)	5.5 [5.4;5.7]	5.7 [5.5;6.0]	7.2 [6.9;7.6]	7.1 [6.8;7.5]	**<0.001**	0.26	0.086
M-value^&^ (µmol/min/kg)	35.3 [30.0;40.6]	38.7 [33.3;44.1]	17.5 [10.3;24.8]	21.6 [14.2;29.0]	**<0.001**	**<0.001**	0.11
HbA_1_c (mmol/mol)	36.9 [35.2;38.6]	34.8 [33.0;36.5]	39.6 [37.3;41.8]	37.5 [35.2;39.9]	0.071	**<0.001**	0.87
HbA1c (%)	5.5 [5.4;5.7]	5.3 [5.2;5.5]	5.8 [5.6;6.0]	5.6 [5.4;5.8]	0.080	**<0.001**	0.90
***Lipid profile***							
FFA_fasting_ (mmol/L)	0.70 [0.62;0.77]	0.62 [0.54;0.69]	0.69 [0.60;0.78]	0.68 [0.59;0.78]	0.86	**0.04**	0.11
Cholesterol (mmol/L)	4.9 [4.6;5.3]	4.4 [4.1;4.7]	4.7 [4.3;5.2]	4.3 [3.9;4.8]	0.44	**<0.001**	0.52
HDL^&^ (mmol/L)	1.4 [1.3;1.5]	1.3 [1.2;1.4]	1.2 [1.1;1.4]	1.1 [1.0;1.2]	0.08	**<0.001**	0.66
LDL (mmol/L)	3.1 [2.9;3.4]	2.8 [2.5;3.1]	2.7 [2.3;3.1]	2.6 [2.2;3.0]	0.09	**<0.001**	0.16
Triglycerides^&^ (mmol/L)	0.94 [0.81;1.11]	0.83 [0.70;0.98]	1.70 [1.38;2.10]	1.50 [1.19;1.90]	**<0.001**	0.08	0.96
***Bone markers***							
Osteocalcin (ng/ml)	7.97 [7.28;8.65]	7.64 [6.92;8.36]	6.64 [5.72;7.55]	6.76 [5.78;7.74]	**0.02**	0.66	0.32
PINP^&^	51.3 [45.7;57.4]	48.1 [42.6;54.3]	38.0 [32.4;44.5]	38.9 [32.9;46.0]	**0.003**	0.68	0.37

All values are model based means [95% confidence intervals]. *P*-value for Baseline indicates the differences between healthy and IR men. *P*-value for Time indicates the change between pre and post measurements in the whole study group. *P*-value for Time*IR interaction indicates if the change in the parameter was different between healthy and IR men.

Abbreviations: BMI, body mass index; FFA, free fatty acids; HbA1c, glycosylated hemoglobin; HDL, high density lipoprotein; IR, insulin resistant; LDL, low density lipoprotein M-value, whole-body insulin sensitivity; PINP, procollagen type 1 N-terminal propeptide; VO_2peak_, aerobic capacity.

^†^Square root or &Logarithmic transformation was performed to fulfill normal distribution assumption.

### Lumbar vertebral region had the highest insulin-stimulated GU

**Figure 3.**
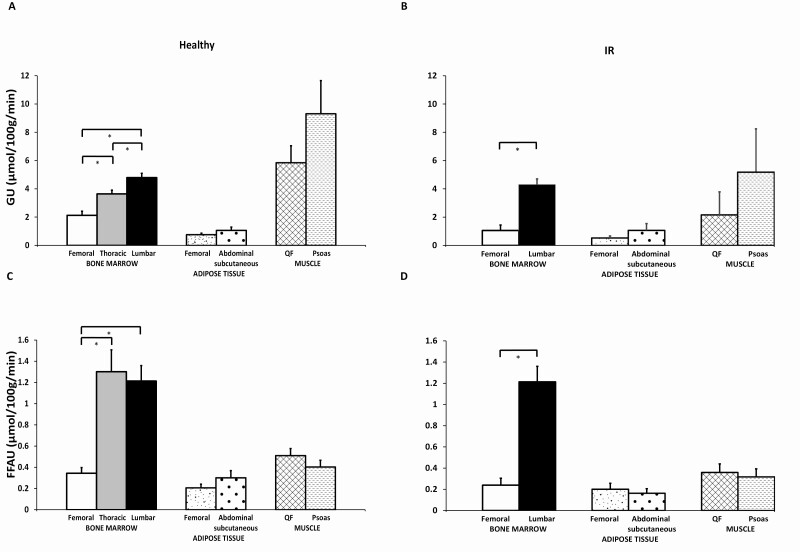
Substrate uptake differs according to anatomic region. Statistical analyses were done only between femoral, thoracic vertebral and lumbar vertebral bone marrow results. *P < 0.0001. Data are model-based means with 95% confidence intervals. Abbreviations: FFAU, free fatty acid uptake; GU, glucose uptake; IR, insulin resistant; QF, quadratus femoris muscle.

At baseline, both bone marrow insulin-stimulated GU (Fig. 3A) and fasting FFAU (Fig. 3B) differed regarding the anatomical region in healthy subjects. Insulin-stimulated GU was significantly higher in lumbar vertebral bone marrow than in thoracic vertebral bone marrow (*P* < 0.0001). Further, GU in femoral bone marrow was significantly lower than GU in lumbar vertebral or thoracic vertebral bone marrow (*P* < 0.0001 for both). Fasting FFAU was higher in lumbar vertebral and thoracic vertebral than in femoral bone marrow (*P* < 0.0001 for both). Similar regional differences in GU (Fig. 3C) and FFAU (Fig. 3D) were observed in the IR group with lumbar vertebral and femoral bone marrow. Thoracic vertebrae were not scanned in the IR group.

### Femoral bone marrow insulin-stimulated GU was impaired in IR subjects and improved after training

At baseline, IR men had higher body mass, body mass index (BMI) and whole-body fat percentage, and their lipid and glucose profiles were impaired (all *P* < 0.001) (Table 1). After the training intervention whole-body fat percentage decreased in the whole group and there were significant improvements in the lipid and glucose profiles (time all *P* < 0.05) (Table 1). No difference was found in the training response between the groups.

**Figure 4.**
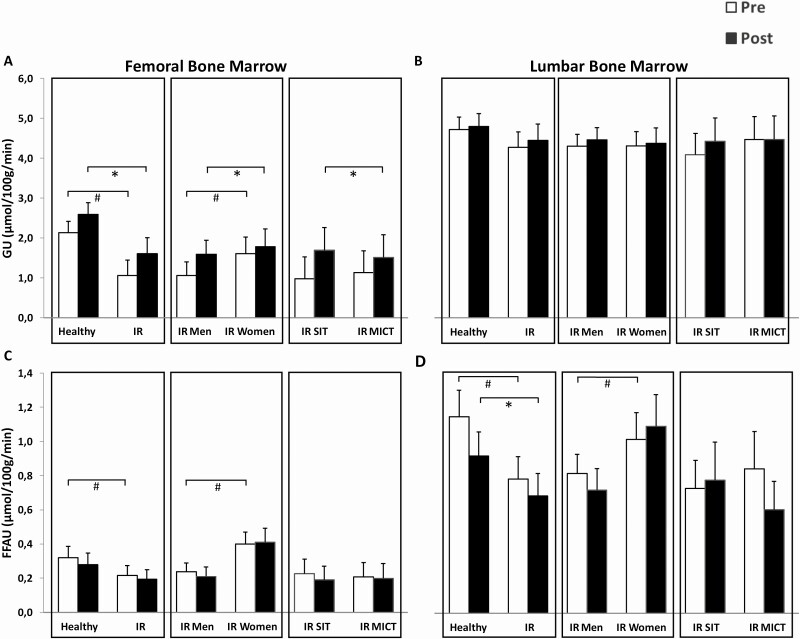
(A-B) Bone marrow insulin-stimulated GU is impaired in IR group and improves after training. (C-D) Bone marrow fasting FFAU is higher in healthy subjects and IR women but improves after training in lumbar vertebrae. # difference at baseline, *P* < 0.05. *difference between pre- and post-measurements. *P* < 0.05. Data are model-based means with 95% confidence intervals. Abbreviations: FFAU, free fatty acid uptake; GU, glucose uptake; IR, insulin resistant; MICT, moderate-intensity continuous training; SIT, sprint-interval training.

Insulin-stimulated GU in femoral bone marrow was impaired in IR men compared to healthy men (*P* < 0.0001). Training improved GU similarly in both groups (Fig. 4A). When femoral muscle GU was included as a covariate, the change in femoral bone marrow GU was no longer significant (data not shown). In lumbar vertebral bone marrow GU, no training induced changes were found in any comparisons (Fig. 4B).

Fasting FFAU in femoral bone marrow was impaired in IR men compared to healthy men (*P* = 0.016) and higher in women compared to men (*P* < 0.001) (Fig. 4C). This same phenomenon can be seen in lumbar vertebral bone marrow (*P* = 0.002 and *P* = 0.023, respectively) (Fig. 4D). Training decreased lumbar vertebral bone marrow FFAU similarly in healthy and IR men. However, no change was seen in comparisons between sexes or training modes.

**Figure 5.**
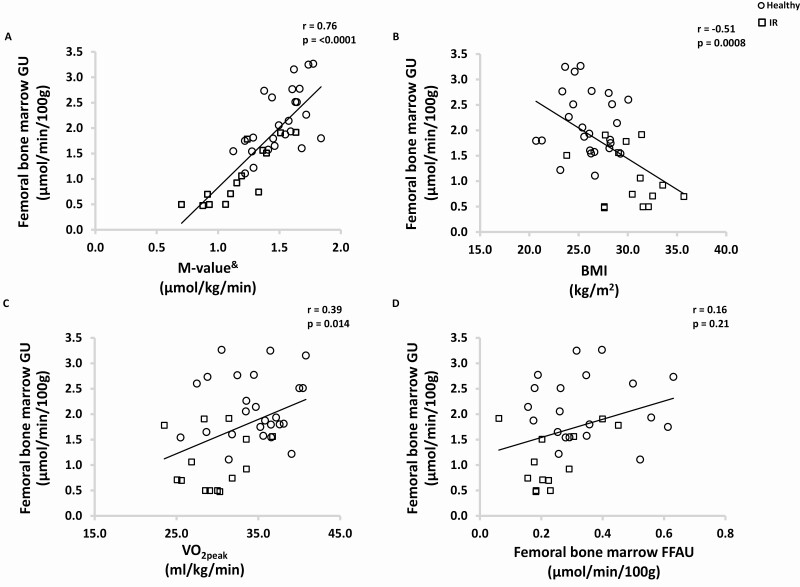
Baseline correlations. Healthy subjects have been marked with a circle and IR subjects with a square. Femoral bone marrow GU correlates positively with whole body insulin sensitivity, aerobic capacity, and negatively with BMI. There was no correlation between femoral bone marrow GU and FFAU. Abbreviations: BMI, body mass index; FFAU, free fatty acid uptake; GU, glucose uptake; IR, insulin resistant; M-value, whole-body insulin sensitivity; VO_2peak_, aerobic capacity. ^&^Logarithmic transformation was performed to fulfill normal distribution assumption.

Femoral bone marrow GU correlated positively with whole-body insulin sensitivity (*P* < 0.0001, r = 0.76, Fig. 5A) and lumbar bone marrow GU (*P* = 0.0004, r = 0.40) and negatively with BMI (*P* = 0.0008, r = −0.51Fig. 5B). Both femoral and lumbar vertebral bone marrow GU correlated positively with aerobic capacity (femoral bone marrow *P* = 0.014, r = 0.39, Fig. 5C and lumbar bone marrow *P* = 0.017, r = 0.38). Femoral bone marrow GU correlated positively with lumbar vertebral bone marrow GU (*P* = 0.0004, r = 0.40) but did not correlate with femoral (Fig. 5D) or lumbar bone marrow FFAU.

A multivariate regression analysis was conducted to study the predictors of femoral and lumbar bone marrow GU and FFAU using the key variables (glycemic status, weight, visceral adipose tissue volume, M-value, VO_2peak_, fasting glucose, osteocalcin, PINP, and bone marrow radiodensity). At baseline, the only statistically significant finding was the association between femoral bone marrow GU and M-value (R^2^ = 0.78, *P* < 0.0001). Also, when we analyzed the change measured after exercise training, M-value still was the only statistically significant predictor for bone marrow GU (R^2^ = 0.59, *P* = 0.0004). None of the aforementioned key variables was a statistically significant predictor for bone marrow FFAU.

### Females had higher femoral and lumbar vertebral FFAU than males

Females had higher body adiposity and level of circulating FFA’s than males at baseline (Table 2). Females had significantly higher femoral and lumbar vertebral bone marrow FFAU at baseline compared to males (Fig. 4C and D). Interestingly, GU differed only for femoral bone marrow (*P* = 0.021, Fig. 4A), with females having higher GU than males. However, training improved femoral bone marrow GU similarly in males and females.

**Table 2.** Subject characteristics between IR men and women before and after exercise intervention

	IR Men		IR Women				
Parameter	Pre	Post	Pre	Post	Baseline	Time	Time*Sex
***Anthropometrics***							
Weight (kg)	96.5 [90.3;102.7]	96.3 [90.1;102.5]	84.3 [76.4;92.2]	83.3 [75.4;91.2]	**0.02**	**0.03**	0.13
BMI (kg/m^2^)	30.5 [29.0;32.0]	30.4 [28.9;31.9]	30.4 [28.5;32.3]	30.0 [28.1;32.0]	0.97	**0.03**	0.10
Whole body fat^&^ (%)	28.5 [26.4;30.8]	27.7 [25.6;29.9]	40.7 [36.8;45.1]	39.4 [35.6;43.6]	**<0.0001**	**0.01**	0.81
Visceral fat^&^ (kg)	4.3 [3.6, 5.2]	4.1 [3.4, 5.0]	2.4 [1.7, 3.2]	2.3 [1.6, 3.1]	**<0.001**	**0.01**	0.83
VO_2peak_ (mL/kg/min)	29.3 [27.4;31.2]	29.9 [28.0;31.9]	23.7 [21.3;26.2]	24.3 [21.7;26.8]	**<0.001**	0.15	0.95
***Glucose profile***							
Glucose_fasting_^&^ (mmol/L)	6.6 [6.2;7.1]	6.6 [6.2;7.1]	6.6 [6.1;7.2]	6.4 [5.8;6.9]	0.95	0.27	0.21
M-value (µmol/min/kg)	17.5 [11.6;23.5]	21.8 [15.6;27.9]	19.9 [12.7;27.0]	22.2 [14.6;29.9]	0.66	0.07	0.59
HBA_1_c (mmol/mol)	39.6 [37.0;42.1]	37.6 [35.0;40.2]	39.5 [36.3;42.8]	37.7 [34.4;41.0]	0.99	**<0.01**	0.88
HbA1c (%)	5.8 [5.5;6.0]	5.6 [5.4;5.8]	5.8 [5.5;6.1]	5.6 [5.3;5.9]	0.99	**0.001**	0.8
***Lipid profile***							
FFA_fasting_ (mmol/L)	0.69 [0.61;0.77]	0.68 [0.60;0.77]	0.96 [0.85;1.07]	0.91 [0.79;1.04]	**<0.0001**	0.38	0.56
Cholesterol (mmol/L)	4.8 [4.3;5.3]	4.4 [3.9;4.9]	5.0 [4.4;5.7]	4.5 [3.9;5.2]	0.50	**0.01**	0.67
HDL^&^ (mmol/L)	1.2 [1.1;1.4]	1.1 [0.9;1.2]	1.5 [1.2;1.7]	1.4 [1.2;1.7]	0.052	**0.02**	0.12
LDL (mmol/L)	2.7 [2.3;3.1]	2.6 [2.2;3.0]	2.9 [2.4;3.5]	2.4 [1.9;3.0]	0.43	**0.01**	0.14
Triglycerides^&^ (mmol/L)	1.7 [1.3;2.2]	1.5 [1.1;2.0]	1.2 [0.9;1.7]	1.2 [0.9;1.7]	0.12	0.55	0.69
***Bone markers***							
Osteocalcin (ng/ml)^&^	6.48 [5.43;7.73]	6.59 [5.51;7.88]	6.12 [4.88;7.67]	6.22 [4.95;7.82]	0.89	0.48	0.99
PINP^&^	37.7 [30.6;46.6]	38.6 [31.3;47.8]	39.1 [30.1;50.8]	37.4 [28.6;48.8]	0.55	0.73	0.27

All values are model based means [95% confidence intervals]. *P*-value for Baseline indicates baseline differences between IR men and women. *P*-value for Time indicates the change between pre and post measurements in the whole study group. *P*-value for Time*Sex interaction indicates if the change in the parameter was different between men and women in the IR group.

Abbreviations: BMI, body mass index; FFA, free fatty acids; HbA1c, glycosylated hemoglobin; HDL, high density lipoprotein; IR, insulin resistant; LDL, low density lipoprotein; M-value, whole-body insulin sensitivity; PINP, procollagen type 1 N-terminal propeptide; VO_2peak_, aerobic capacity.

^&^Logarithmic transformation was performed to fulfill normal distribution assumption.

### Radiodensity was higher in lumbar vertebral bone marrow than femoral bone marrow

Radiodensity was higher in lumbar vertebral bone marrow (186.1 HU) than in femoral bone marrow (81.0 HU) in men (*P* < 0.0001). Femoral bone marrow radiodensity was significantly lower in healthy men (74.5 HU) compared to IR men (87.5 HU, *P* = 0.035). There was no difference in lumbar vertebral bone marrow radiodensity between healthy (191.6 HU) compared to IR subjects (180.5 HU, *P* = 0.35). There were no exercise induced changes in any of the groups. There was no significant difference between femoral or lumbar vertebral bone marrow radiodensity between IR men and women. Femoral bone marrow radiodensity correlated positively with weight (*P* = 0.009, r = 0.29), BMI (*P* = 0.016, r = 0.27), fasting glucose (*P* = 0.023, r = 0.27), and fasting FFA (*P* = 0.025, r = 0.26) and correlated inversely with femoral bone marrow insulin stimulated GU (*P* = 0.035, r = −0.25). Lumbar vertebral bone marrow radiodensity correlated negatively with age (*P* = 0.031, r = −0.25) and whole body fat percentage (*P* = 0.025, r = −0.25) and positively with VO_2peak_ (*P* = 0.002, r = 0.34).

### Bone formation and remodeling markers were lower in the IR group

**Figure 6.**
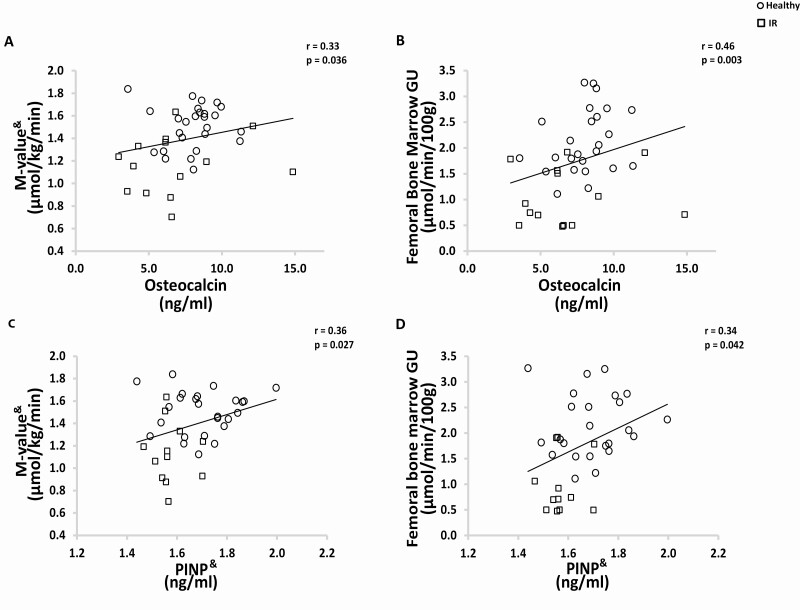
Bone turnover marker correlations at baseline. Healthy subjects have been marked with a circle and IR subjects with a square. (A-B) Osteocalcin correlates positively with whole-body insulin sensitivity and femoral bone marrow GU. (C-D) Also PINP correlates positively with whole-body insulin sensitivity and femoral bone marrow GU. Abbreviations: GU, glucose uptake; IR, insulin resistant; M-value, whole-body insulin sensitivity; PINP, procollagen type 1 N-terminal propeptide. ^&^Logarithmic transformation was performed to fulfill normal distribution assumption.

At baseline, plasma osteocalcin concentration was lower in the IR than in the healthy group (*P* = 0.021; Table 1). Osteocalcin concentration correlated positively with whole-body insulin sensitivity (*P* = 0.036; Fig. 6A) and femoral bone marrow GU (*P* < 0.003, Fig. 6B). At baseline, PINP concentration was also lower in the IR than in the healthy group (*P* = 0.003; Table 1). PINP concentration correlated positively with whole-body insulin sensitivity (*P* = 0.027; Fig. 6C), femoral bone marrow GU (*P* = 0.042; Fig. 6D), femoral bone marrow FFAU (*P* = 0.027, r = 0.37), and lumbar vertebral bone marrow FFAU (*P* = 0.026, r = 0.37), and correlated negatively with BMI (*P* = 0.033, r = −0.34) and blood triglycerides (*P* = 0.017, r = −0.37). However, exercise training had no effect on osteocalcin or PINP concentrations in either group (Table 1).

## Discussion

Here we show that there are differences in bone marrow metabolism depending on the anatomical location. Furthermore, bone marrow metabolism is impaired in IR and can be improved by exercise training. A 2-week exercise training intervention increased bone marrow insulin-stimulated GU and decreased FFAU both in healthy and IR subjects. In IR subjects GU and FFAU were higher in females compared to males. Femoral bone marrow GU correlated positively with aerobic capacity and whole-body insulin sensitivity, and negatively with BMI. At baseline, osteocalcin and PINP levels were lower in the IR compared to healthy group and correlated with femoral bone marrow GU but were not affected by exercise training.

This study shows that insulin-stimulated GU in healthy humans differs according to anatomical location between thoracic vertebral, lumbar vertebral, and femoral bone marrow, being highest in lumbar vertebral and lowest in femoral bone marrow. Fasting FFAU was also higher in vertebral than femoral bone marrow. The same phenomenon can be seen in the IR group in lumbar vertebral and femoral bone marrow. This may be explained by the differences in the composition of bone marrow and its role in hematopoiesis. In adult humans, the middle of the thigh is mostly adipose tissue, whereas the lumbar vertebral region still actively produces blood cells (1,2). The need for energy of hematopoietic tissue seems to be constant and not easily affected by environmental factors. However, femoral bone marrow appears to serve mainly as a fat depot, which has been shown to be insulin sensitive and react to exercise (11). When we further tested this hypothesis using CT-derived HU, the radiodensity was lower in femoral than lumbar vertebral bone marrow indicating higher fat content in femoral bone marrow. However, the bone marrow cavity includes adipose tissue, hematopoietic tissue, and trabecular bone. Therefore, attenuation measurements of bone marrow cavity include multiple tissues and adipose tissue alone cannot be quantified. Nevertheless, the lower the HU, the higher the fat content (20), and in this context, we found that lumbar vertebral bone marrow radiodensity was in inverse relationship with age and whole-body fat percentage suggesting either an increase in adiposity or decrease in trabecular bone in lumbar bone marrow cavity with aging and obesity whereas in femoral bone marrow cavity, where the influence of trabecular bone on bone marrow radiodensity is negligible, we found that lower femoral bone marrow radiodensity (high-fat content) in healthy compared to IR subjects. Further, we found that there exists a direct correlation of femoral bone marrow radiodensity with body weight and BMI and an inverse relationship with insulin-stimulated femoral bone marrow GU. These findings are in line with Ermitici et al (24), where bone marrow fat content was measured using proton magnetic resonance spectroscopy, and it was found that bone marrow fat content (%) was inversely related to the index of whole-body IR. It is possible that a higher fat content (more adipocytes) in bone marrow in healthy compared to IR subjects drive an increase in insulin-stimulated GU since marrow adipocytes express insulin receptors (25), and previously we have also shown that insulin stimulates GU in bone marrow adipose tissue (26). In line with these findings femoral bone marrow GU, but not lumbar vertebral bone marrow, correlated negatively with BMI and positively with whole-body insulin sensitivity, and IR subjects had lower insulin-stimulated bone marrow GU than healthy subjects at baseline.

After the training we found an increase in femoral bone marrow insulin-stimulated GU but not in lumbar vertebral and thoracic vertebral bone marrow. In femoral bone marrow, training increased GU in all groups. This finding agrees with our previous data from the same study protocol regarding the changes in skeletal muscle GU where we showed that GU improved only in the working muscles of the lower extremities and not in the upper body muscles (13). The training intervention consisted of bicycle ergometer training, which mainly strains the lower extremities, explaining why GU improved only in femoral bone marrow. Our data agree also with the findings of Huovinen and colleagues, who investigated the effects of whole-body resistance training on insulin-stimulated bone marrow in elderly subjects and found that GU improved in femoral bone marrow but not in vertebral bone marrow after training (11). The difference in training response between vertebral and femoral bone marrow GU could also be explained by the amount of fat and the role of bone marrow. Hematopoietic tissue in lumbar vertebral bone marrow may not be as easily affected by environmental factors compared to femoral bone marrow.

Increase in the femoral bone marrow insulin-stimulated GU was no longer significant when we corrected the statistical analysis for muscle GU. This suggests that the increase in femoral bone marrow GU was not independent of the increase of GU in the surrounding muscle tissue. Indeed, in the multivariate analysis M-value showed to be the only statistically significant predictor of bone marrow insulin-stimulated GU. Our finding of increased femoral bone marrow GU may be also partially due to the PET methodology related spillover effect—that is, spilling of activity from the neighboring high activity tissues (muscles) to the less active areas (bone tissue). However, cortical bone between bone marrow cavity and muscle tissue should minimize the spillover effect (Fig. 2). Our study suggests that bone marrow metabolism is improved by exercise training, however, further studies are needed to clarify the proportion of independent and muscle metabolism-induced changes.

The comparisons between males and females were made within the IR group. We found that females had higher femoral bone marrow GU and higher femoral and lumbar vertebral FFAU than males at baseline. Females are known to be more insulin sensitive than men (27,28), and our results show this also at bone marrow level. At baseline, females had higher amount of circulating FFAs than males, which explains the difference in FFAU. Exercise training had no effect on the amount of circulating FFAs. Also, FFAU was not affected by exercise training in the IR group. The sample size for IR men and women was small so this response should be investigated further.

Osteocalcin is a biochemical bone formation marker that is produced by osteoblasts. Osteocalcin concentrations were lower in IR than in healthy group at baseline in the present study, which is in line with previous observations (29). It has also been shown before that circulating osteocalcin is negatively associated with IR, obesity, and diabetes (30). Our results support these findings, as osteocalcin correlated positively with whole-body insulin sensitivity. In addition, to our knowledge, we show here for the first time that osteocalcin correlates positively with femoral bone marrow GU at baseline. PINP is synthesized by osteoblasts as part of Type I collagen formation, and it has been recommended to be used as a reference analyte for bone turnover markers in observational and intervention studies (22). It has not been clearly established yet how IR or diabetes affects PINP concentration (31). Similarly to osteocalcin, PINP concentration was lower in the IR group than in the healthy group. PINP also correlated positively with bone marrow GU and FFAU, whole-body insulin sensitivity, and negatively with BMI. However, there were no significant changes in either of the bone markers after the exercise training intervention. A 2-week training intervention may be too short to induce significant changes in circulating osteocalcin or PINP concentrations. Also, training consisted of cycling, which may not stimulate bone turnover as much as, for example, running or other high-impact exercise (32, 33).

This study is not without limitations. The number of study subjects was relatively small but typical for exercise training trials using demanding molecular imaging modalities. To avoid spillover effect from surrounding tissues, ROIs were drawn carefully in the bone marrow cavity on the PET images. Cortical bone acts as a barrier between bone marrow and surrounding tissue, so it is unlikely that spillover from muscles could have affected the bone marrow results. To stimulate bone marrow metabolism and bone turnover optimally, running or other high-impact loading exercise would probably have been the best type of exercise. However, to standardize the SIT and MICT protocols in laboratory settings in sedentary subjects, we preferred cycling in the current study. These findings show only the early training response, and the long-term effects of these training modes on bone marrow metabolism should be studied in further experiments.

## Conclusion

Our data suggest that bone marrow metabolism differs regarding anatomical location and is impaired in IR. We show for the first time that short-term exercise training improves bone marrow glucose and free fatty acid metabolism similarly in healthy and IR men, similarly in men and women and regardless of training method. We also show that bone turnover markers osteocalcin and PINP are associated with insulin sensitivity.

## Data Availability

The data sets generated during and/or analyzed during the current study are not publicly available but are available from the corresponding author on reasonable request.

## Additional Information

***Disclosure Summary:*** The authors have nothing to disclose.

## References

[1] Kricun ME . Red-yellow marrow conversion: its effect on the location of some solitary bone lesions. Skeletal Radiol. 1985;14(1):10-19. 3895447 10.1007/BF00361188

[2] Vogler JB 3rd, Murphy WA. Bone marrow imaging. Radiology. 1988;168(3):679-693. 3043546 10.1148/radiology.168.3.3043546

[3] Fazeli PK, Horowitz MC, MacDougald OA, et al. Marrow fat and bone–new perspectives. J Clin Endocrinol Metab. 2013;98(3):935-945. 23393168 10.1210/jc.2012-3634 PMC3590487

[4] Shanbhogue VV, Mitchell DM, Rosen CJ, Bouxsein ML. Type 2 diabetes and the skeleton: new insights into sweet bones. Lancet Diabetes Endocrinol. 2016;4(2):159-173. 26365605 10.1016/S2213-8587(15)00283-1

[5] Schwartz AV . Epidemiology of fractures in type 2 diabetes. Bone. 2016;82:2-8. 26027505 10.1016/j.bone.2015.05.032

[6] Napoli N, Chandran M, Pierroz DD, Abrahamsen B, Schwartz AV, Ferrari SL; IOF Bone and Diabetes Working Group. Mechanisms of diabetes mellitus-induced bone fragility. Nat Rev Endocrinol. 2017;13(4):208-219. 27658727 10.1038/nrendo.2016.153

[7] Zhu M, Hao G, Xing J, et al. Bone marrow adipose amount influences vertebral bone strength. Exp Ther Med. 2019;17(1):689-694. 30651851 10.3892/etm.2018.7003 PMC6307407

[8] Shen W, Chen J, Gantz M, et al. MRI-measured pelvic bone marrow adipose tissue is inversely related to DXA-measured bone mineral in younger and older adults. Eur J Clin Nutr. 2012;66(9):983-988. 22491495 10.1038/ejcn.2012.35 PMC3396793

[9] Schellinger D, Lin CS, Hatipoglu HG, Fertikh D. Potential value of vertebral proton MR spectroscopy in determining bone weakness. AJNR Am J Neuroradiol. 2001;22(8):1620-1627. 11559519 PMC7974571

[10] Naveiras O, Nardi V, Wenzel PL, Hauschka PV, Fahey F, Daley GQ. Bone-marrow adipocytes as negative regulators of the haematopoietic microenvironment. Nature. 2009;460(7252):259-263. 19516257 10.1038/nature08099 PMC2831539

[11] Huovinen V, Bucci M, Lipponen H, et al. Femoral bone marrow insulin sensitivity is increased by resistance training in elderly female offspring of overweight and obese mothers. PLoS One. 2016;11(9):e0163723. 27669153 10.1371/journal.pone.0163723 PMC5036877

[12] Honkala SM, Motiani KK, Eskelinen JJ, et al. Exercise training reduces intrathoracic fat regardless of defective glucose tolerance. Med Sci Sports Exerc. 2017;49(7):1313-1322. 28628064 10.1249/MSS.0000000000001232 PMC5473372

[13] Eskelinen JJ, Heinonen I, Löyttyniemi E, et al. Muscle-specific glucose and free fatty acid uptake after sprint interval and moderate-intensity training in healthy middle-aged men. J Appl Physiol (1985). 2015;118(9):1172-1180. 25767035 10.1152/japplphysiol.01122.2014

[14] Heiskanen MA, Motiani KK, Mari A, et al. Exercise training decreases pancreatic fat content and improves beta cell function regardless of baseline glucose tolerance: a randomised controlled trial. Diabetologia. 2018;61(8):1817-1828. 29717337 10.1007/s00125-018-4627-x PMC6061150

[15] Motiani KK, Savolainen AM, Toivanen J, et al. Effects of short-term sprint interval and moderate-intensity continuous training on liver fat content, lipoprotein profile, and substrate uptake: a randomized trial. J Appl Physiol (1985). 2019;126(6):1756-1768. 30998125 10.1152/japplphysiol.00900.2018 PMC6620664

[16] American Diabetes Association. Classification and diagnosis of diabetes. Diabetes Care 2015;38(Suppl 1):S8-S16. 10.2337/dc15-S005 25537714

[17] Motiani KK, Savolainen AM, Eskelinen JJ, et al. Two weeks of moderate-intensity continuous training, but not high-intensity interval training, increases insulin-stimulated intestinal glucose uptake. J Appl Physiol (1985). 2017;122(5):1188-1197. 28183816 10.1152/japplphysiol.00431.2016 PMC5451533

[18] DeFronzo RA, Tobin JD, Andres R. Glucose clamp technique: a method for quantifying insulin secretion and resistance. Am J Physiol. 1979;237(3):E214-E223. 382871 10.1152/ajpendo.1979.237.3.E214

[19] Goldman LW . Principles of CT and CT technology. J Nucl Med Technol. 2007;35(3):115-28; quiz 129. 17823453 10.2967/jnmt.107.042978

[20] Singhal V, Bredella MA. Marrow adipose tissue imaging in humans. Bone. 2019;118:69-76. 29331301 10.1016/j.bone.2018.01.009 PMC6039291

[21] Kiviniemi AM, Tulppo MP, Eskelinen JJ, et al. Cardiac autonomic function and high-intensity interval training in middle-age men. Med Sci Sports Exerc. 2014;46(10):1960-1967. 24561814 10.1249/MSS.0000000000000307

[22] Vasikaran S, Cooper C, Eastell R, et al. International Osteoporosis Foundation and International Federation of Clinical Chemistry and Laboratory Medicine position on bone marker standards in osteoporosis. Clin Chem Lab Med. 2011;49(8):1271-1274. 21605012 10.1515/CCLM.2011.602

[23] Paldánius PM, Ivaska KK, Hovi P, et al. The effect of oral glucose tolerance test on serum osteocalcin and bone turnover markers in young adults. Calcif Tissue Int. 2012;90(2):90-95. 22147278 10.1007/s00223-011-9551-8

[24] Ermetici F, Briganti S, Delnevo A, et al. Bone marrow fat contributes to insulin sensitivity and adiponectin secretion in premenopausal women. Endocrine. 2018;59(2):410-418. 28624865 10.1007/s12020-017-1349-7

[25] Lecka-Czernik B . Marrow fat metabolism is linked to the systemic energy metabolism. Bone. 2012;50(2):534-539. 21757043 10.1016/j.bone.2011.06.032 PMC3197966

[26] Pham TT, Ivaska KK, Hannukainen JC, et al. Human bone marrow adipose tissue is a metabolically active and insulin-sensitive distinct fat depot. J Clin Endocrinol Metab 2020;105(7):1-11. 10.1210/clinem/dgaa216 PMC7247553 32311037

[27] Kautzky-Willer A, Brazzale AR, Moro E, et al. Influence of increasing BMI on insulin sensitivity and secretion in normotolerant men and women of a wide age span. Obesity (Silver Spring). 2012;20(10):1966-1973. 22282046 10.1038/oby.2011.384

[28] Yki-Järvinen H . Sex and insulin sensitivity. Metabolism. 1984;33(11):1011-1015. 6387364 10.1016/0026-0495(84)90229-4

[29] Ivaska KK, Huovinen V, Soinio M, et al. Changes in bone metabolism after bariatric surgery by gastric bypass or sleeve gastrectomy. Bone. 2017;95:47-54. 27818311 10.1016/j.bone.2016.11.001

[30] Addai D, Zarkos J, Tolekova A. The bone hormones and their potential effects on glucose and energy metabolism. Endocr Regul. 2019;53(4):268-273. 31734651 10.2478/enr-2019-0027

[31] Costantini S, Conte C. Bone health in diabetes and prediabetes. World J Diabetes. 2019;10(8):421-445. 31523379 10.4239/wjd.v10.i8.421 PMC6715571

[32] Beck BR, Daly RM, Singh MA, Taaffe DR. Exercise and Sports Science Australia (ESSA) position statement on exercise prescription for the prevention and management of osteoporosis. J Sci Med Sport. 2017;20(5):438-445. 27840033 10.1016/j.jsams.2016.10.001

[33] Heinonen A, Kannus P, Sievänen H, et al. Randomised controlled trial of effect of high-impact exercise on selected risk factors for osteoporotic fractures. Lancet. 1996;348(9038):1343-1347. 8918277 10.1016/S0140-6736(96)04214-6

